# Application of structural equation modelling to develop a conceptual model for smallholder’s credit access: The mediation of agility and innovativeness in organic food value chain finance

**DOI:** 10.1371/journal.pone.0235921

**Published:** 2020-08-04

**Authors:** Ali Abid, Shang Jie, Waqas Aslam, Saima Batool, Yue Lili

**Affiliations:** 1 College of Economics and Management, Agricultural Economics and Management, Northeast Forestry University, Xiangfang District, Harbin, Heilongjiang, China; 2 School of Management, Tourism & Hotel Management, Zhejiang University, Zhejiang, Hangzhou, China; 3 Resources and Development of Traditional Chinese Medicine, Heilongjiang University of Traditional Chinese Medicine, Harbin, Heilongjiang, China; Univerza v Mariboru, SLOVENIA

## Abstract

Developing a conceptual model is vital for small-scale organic farmer’s credit access to sustain the livelihoods. However, smallholders continually face severe problems in getting finance that lead to reduce investment and in turn, challenges the livelihoods. Therefore, the aim of the present study was to establish and empirically test a theoretical model to explore how agility and innovativeness in organic food value chain finance are achieved through ITI, TRST, CG, ICT, and IS, and how these, in turn, can accelerate financial flow in the value chain and enhance competitiveness. The present study used a survey method and collected data from small-scale farmers, traders, and financial institutions. The model and hypothesis are tested using data obtained from 331 respondents through partial least square structure equation modeling techniques. We argue that development of theoretical model show potential to increase creditworthiness of smallholders and overcome uncertainties that impede traditional value chain credit arrangement. Thus, the present study could provide new ways to integrate the value chain partners, through information and communication technology and governance arrangements in the organic food value chain financing. This study demonstrates that the mediations of innovativeness and agility significantly affect the development of new financial products to make agile the financial flow, which in turn positively influences value chain competitiveness. Significant judgments are required for trustworthy relations among the value chain partners to positively harness innovative product development for swifter value chain finance. Therefore, this theoretical model should not be regarded as a quick solution, but a process of testing, error, and learning by doing so.

## Introduction

Wheat is the major crop of Pakistan, which is cultivated by 80% of farmers, comprising an area of approximately 9 million hectares, which is almost 40% of the entire cultivated area [1]. Recently, an increase in organic farming has created an opportunity for small-scale farmers of wheat, due to the high cost of synthetic fertilizer and the demand for organic food [2]. The National Institute of Organic Agriculture, Government of Pakistan, has strongly supported organic food production methods that have been certified by Zwolle, Netherlands, in adherence to (EEC NO. 2092/91) and USDANOP standards [3,4]. Organic wheat output in Pakistan is dominated by smallholder farmers, which provides a high return on investment and makes a significant contribution to farm incomes [4]. In the markets, organic producers either sell their produce at specified outlets or to larger certified firms [5, 6]. This suggests the critical relevance of wheat farming to the livelihood of small-scale farmers as well as the significance of organic food production.

Nonetheless, smallholder organic producers continually face severe problems in getting finance that lead to reduced investment [3]. Apart from insufficient financial investments, the challenges of these farmers’ livelihood, are also stated which need to be addressed at both the farm and national policy levels [7]. Access to finance by smallholder farmers is therefore very important to improve farmers’ livelihoods. Investment, access to credit, and financial support are critical factors for the development of hazard-free, green, and organic production [8–11], thus offering hope to improve farmers’ livelihoods. Even with the development of the banking industry, smallholders’ access to finance has proved to be a challenge in developing countries. Currently, access to finance is incredibly low in the rural areas of Pakistan and 6% of poor rural smallholders are banked [12]. Particularly in Pakistan, this lack of bank loan penetration is due to geographically dispersed areas [13], lack of information, knowledge, and guarantee or collateral [14,15]. This is because access to financial services is a risk for small-scale organic farmers. However, banks are unaware of the organic production system and face difficulty in assessing credit eligibility. As a result, financial services of banks are very limited to organic producers and it is very difficult for smallholders to get loans [16, 17]. In addition, the rural low-income smallholders have little formal relationship with the banking system; this makes them dependent on arthi (middlemen) for informal loans, who charge predatory prices for loans because of their low creditworthiness [18,19]. According to Haq et al. [20] and Altenbuchner et al. [19] arthi appears to be a service provider fulfilling the financial needs of farmers in rural areas where the formal financial sector is not considered creditworthy. Even though the positive attributions of arthi, the previous studies on smallholder credit access, have shown that these middlemen earned much more money than the banks or formal institutes [21]. This is because of the diverse and multidisciplinary challenges of farmers’ livelihood systems and the kind of finance to be addressed [22]. The growing challenges for smallholder farmers' credit point to adopting methods that are easy to obtain credit for, increase their productivity and are helpful in market access [16,23]. In other words, we look at it from the perspective of the value chain.

The value chain approach addresses the challenges holistically and mostly considers the competitiveness and risk management aspect of the entire chain. Therefore, the value chain for financial institutions can play a very effective role as it will reduce the transaction cost and associated risks with microcredit, which will in turn not only increase the credibility of the small farmers but also strengthen the financial services to the chain partners [24,25]. However, the associated risk such as the breach of re-payment commitments, led to mistrust among value chain partners [16,26]. This is because value chains reflect dynamic processes among various actors with different points of view [27], so the small changes in supply and demand for organic products lead to intended and unintended outcomes [17]. Therefore, trust-based partnership is needed to sustain competitiveness in the organic market, even if there is interdependence among value chain partners [28]. This is however challenging in smallholder farming contexts in Pakistan where outcomes are usually unpredictable [3] Thus, in today's era, it is important to find ways by which these barriers to collaboration, trust and risk might be overcome.

In the light of this, advancements in value chain-based model offers significant advantages to develop producers-buyers strategic partnership for acceleration of financial flow. A business model is defined as a way in which the activities of value chain actors are being organized [29]. A business model, therefore, organizes the value chain actors in order to reduce transaction costs, information sharing facilities to avoid asymmetric information, and innovative financial products to manage risks along the chain [30]. In the past, several studies have developed financing models to strengthen and extend financial products and services to the agricultural sector [24,30–35]. Previous studies on agricultural value chain finance, have identified that chain integration, strategic partnering, supporting services and product range related factors influence the value chain competitiveness [31]. Interestingly, studies on value chain finance are lacking in the main literature on organic farming. To our knowledge, no prior studies have developed a model in organic food value chain finance. This affects the sustainability of the organic food business which in turn risks the livelihoods of small-scale farmers. It is therefore critical to develop a conceptual model for small’ holders credit access in organic food value chain. Therefore, the objective of this paper is to address the following questions; (1) to develop a model to increase creditworthiness of smallholders; and (2) what critical factors should be considered to mitigate risk in organic food value chain finance?

To address these questions, the present study utilizes the partial least square structural equation modelling to quantify critical factors. The paper is comprised of five sections, the first of which is based on an introduction. The second part is based on literature review. The third section is about theoretical background and hypothesis development. The fourth section discusses the research method. The fifth section contains the analysis and results of the research work. The last section discusses the results of our study and its practical contributions.

## Literature review

Several studies have explored that value chain financing plays a vital role in providing finance that are needed at different stages of production and processing [36]. A study done by Oberholster and his research fellows, have identified the major factors, such as, chain integration, strategic partnering, supporting services and product range that influence the value chain competitiveness. They determined that development of conceptual model has the ability to build strong relationships between financial institutions and small-scale producers [31]. A study of Miller and Jones [25] have found that value chain finance offers an opportunity to reduce cost and risk in financing, and reach out to smallholder farmers. Kopparthi and Kagabo [37] have conducted direct interviews with 122 farmers and staff of the financial institutions in Rwanda. They determined that value chain financing improved the productivity and profits of farmers. Some other studies have found the key contribution of technology and information sharing in value chain finance that not only increase the production efficiency but also reduce the transaction cost [30]. Further, they also determined that innovations in the products have the ability to integrate smallholders and reduce the transaction costs. The study of Mahajan and Gouri [38] have found that development of financing models, have the capability to integrate the value chain actors through contract arrangements that significantly reduce the asymmetric information and increase the financial flow within value chain. From the scientific literatures, it has been revealed that most of the research was based on the case studies by using qualitative methods and most of the information obtained through questionnaire survey [36, 30, 38]. Nevertheless, some of the researchers used quantative approach and conducted exploratory factor analysis by using LISREL software [31].

However, none of the study has used the partial least square structural equation modelling technique. PLS-SEM is a very effective technique for analyzing the cause-effect relationships between variables [39]. It is a growing multivariate analysis technique for calculating variance-based structural equation models, especially in the field of social sciences [40]. More recently, several studies in social sciences have employed PLS-SEM to resolve complex relationships that are otherwise difficult to disclose. For example, Issa and Hamm [41] have investigated the positive attitude and intentions of Syrian farmers to adopt organic farming. Pappa et al. [42] have proposed a model and identified the factors that influence farmers' and processors' behavior regarding the installation and operation of an electronic traceability system. Yaseen et al. [43] have employed PLS-SEM and explored that market orientation fosters farmers’ ability to create value within commodity markets in Kenya. By applying the PLS-structural equation modelling, Guo et al. [44], have examined the interacting mechanisms of livelihood capital, livelihood strategy, and agricultural land transfer in the hilly areas of Sichuan, China. Karimi and Sotoodeh [45] have employed PLS-SEM, to examine the mediating role of intrinsic motivation in student’s academic engagement by using a sample of 365 agriculture students in Iran.

Therefore, in this paper a model has yet to be established to understand the factors that may increase the credit access of smallholder farmers and mitigate the risk in organic food value chain finance. Therefore, the present study surveyed the factors that would be useful for the creditworthiness of organic farmers, and applied an advanced multivariate analysis technique of the PLS path model using Smart-PLS software to examine and validate the conceptual model.

## Conceptual model and hypothesis development

**Fig 1:**
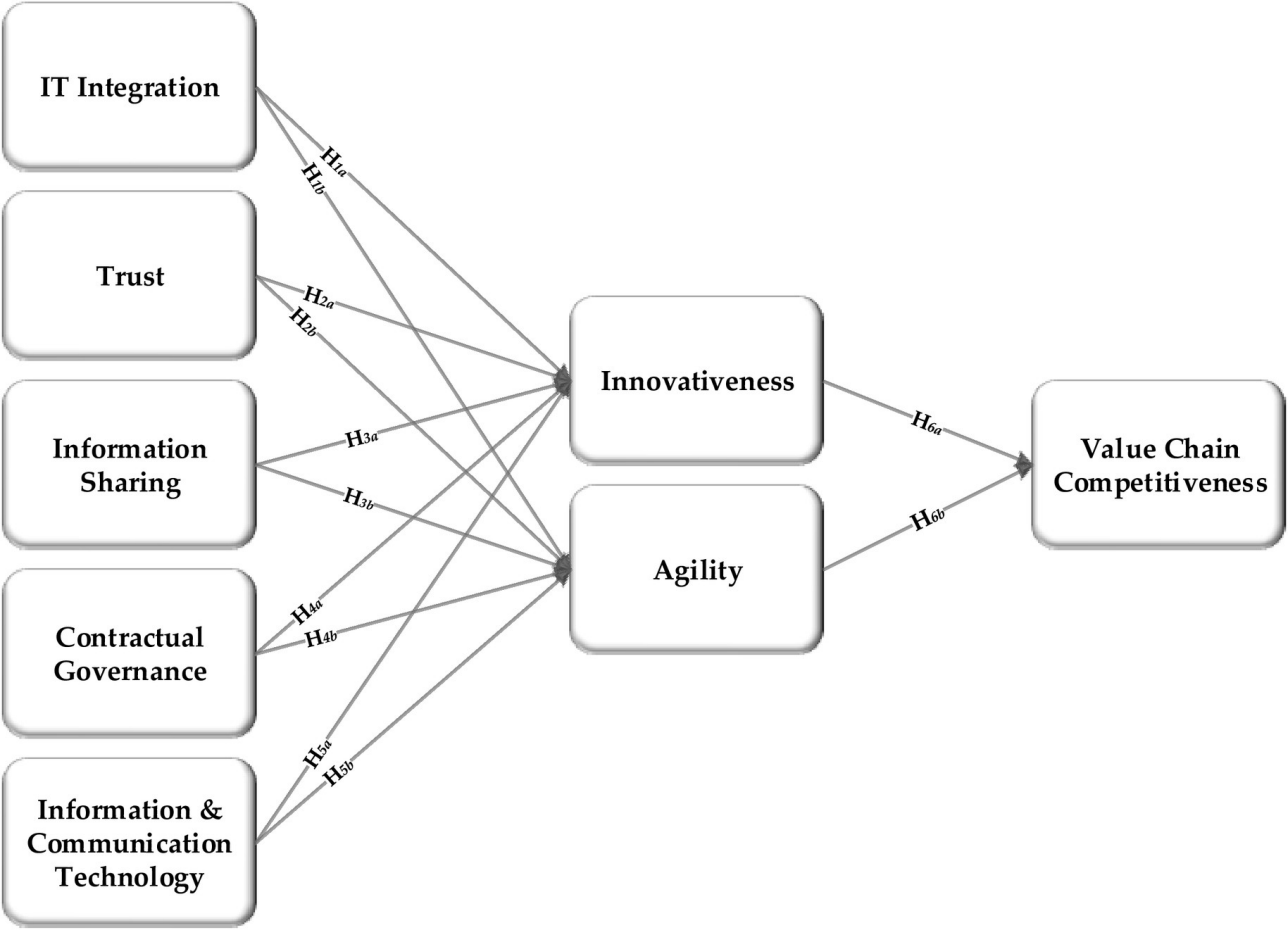
Conceptual model.

**Fig 2:**
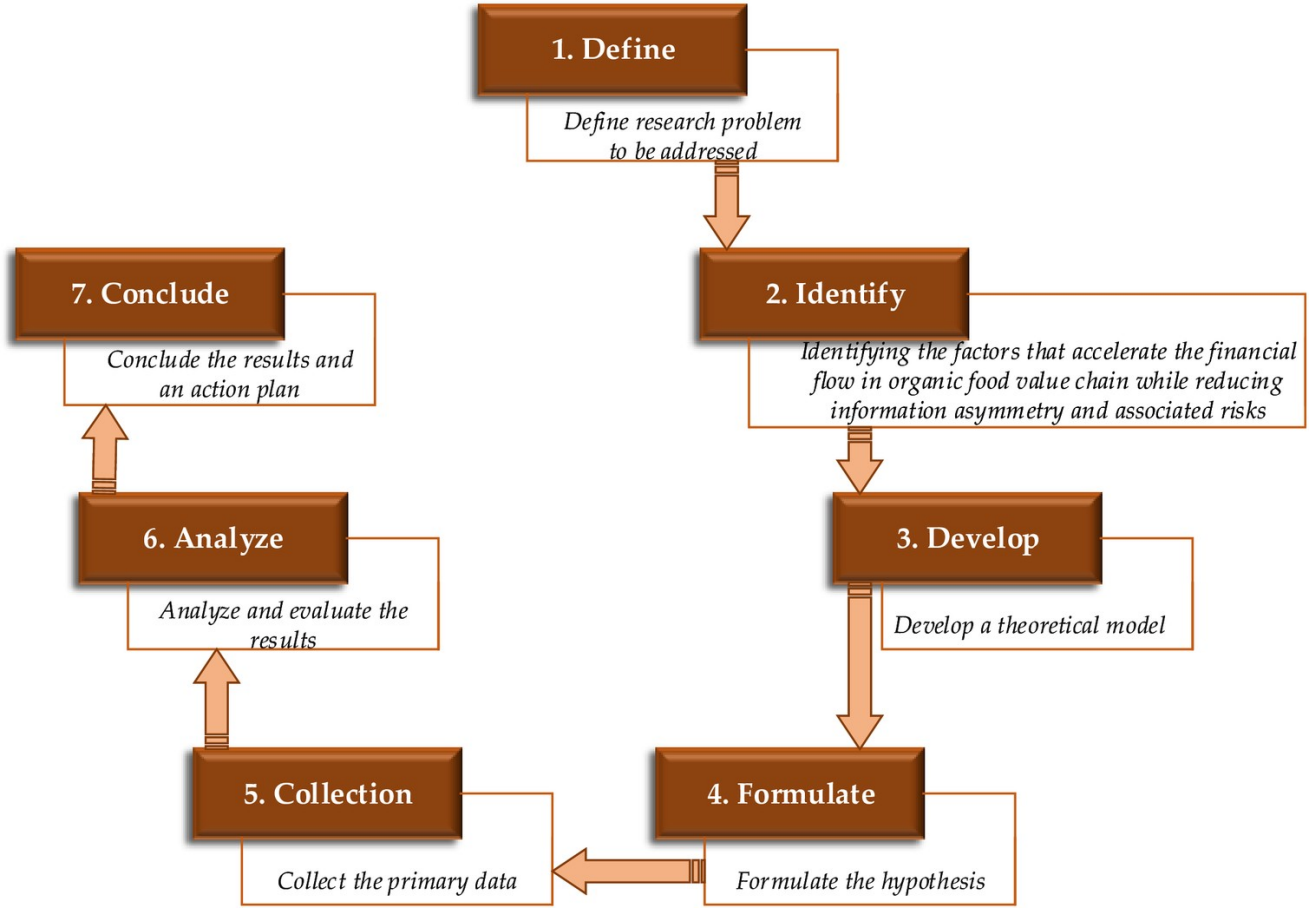
Research steps.

In this paper, a conceptual model has been developed through an examination of scientific literature (Fig 1) and tested through partial least square structural equation modelling. The purpose of model is to address the needs smallholders by organizing value chain actors, and what factors should be considered to reduce transaction cost and manage risks in organic food value chain finance. To address this, innovativeness and agility are seemed to be important determinants to address the financial needs of customers [46]. Besides these determinants, trust also seemed to be an effective and permanent attractive feature to cope with confusion and handle risky situations among value chain partners [47]. Through effective information sharing [48] and quick interaction with ICT [49], contractual governance among members in value chains [50]; financial institutes can further develop innovative products to satisfy the diversified needs of small-scale producers [25,51,52]. A series of steps, starting with defining a research problem to writing a research paper are presented in (Fig 2).

### IT integration

ITI has long been theorized to prevent unnecessary information, lower the confusion, enhance the efficiency of information processing, and create an effective communication environment among partners by decreasing information asymmetries [53]. Based on valid information, partners and stakeholders of the value chain can make sound judgments in this way; thus, ITI plays a key role in VCF. ITI is known as a degree of collaboration and the exchange of relevant information among value chain partners [54].

Organic food production is rapidly growing all over the world. This change presents opportunities for smallholders to become integrated into a value chain [55–57]. The integrated value chain has the ability to revitalize local food economies, avoiding informational asymmetry and enhancing farmers productivity and market access [58]. Interaction through ITI will strengthen the creditworthiness of smallholder farmers. IT integration between producer and buyer has the ability to reduce transaction cost and allowed financial services provider to be more efficient. Therefore, financial institutions can augment their services through IT integration and swifter the value chain finance than ever before [25,29,30]. ITI is helpful in addressing and fixing the problems before their occurrence as well as creating innovative solutions for sustainable financial flow. Hence, the integration of the organic food value chain through IT, make agile financial flows and improve competitiveness.

### Trust

Trust refers to the extent to which value chain partners consider each other reliable and credible. Trust is an important factor in reducing uncertainties and tackling unpredictable circumstances in a community where multifaceted outcomes are possible [59]. According to Luhmann, when there is no trust left in a society, the rational action becomes limited and the partners restrict or withdraw their activities [60]. Trust is therefore considered as an important element in bridging unpredictable situations and acting in perceived risky situations [61]. Previous studies have found that trust among partners is an essential requirement for successful management of financial flow in the value chain [62].

The relationship between consumers and producers is of great importance for building trust in the value chain. This is because consumers' participation in organic food value chains builds trust relationships with organic producers and reduces information asymmetry [63]. According to Miller, trust between the producer and the buyer is one of the key factors that can drive innovation in value chain finance and continue to mitigate risks for lending institutes [25]. Through these innovations, the financial institute has the ability to integrate the smallholders and reduce their transaction costs [64,65]. Hence, the implementation of an agile VCF relies on a higher degree of trust [66].

### Information sharing

Information sharing describes how the partners of the value chain interact with each other over time with effective, accurate, complete, and confidential information. The sharing of information is of great significance for a number of reasons; it may increase the degree of competitiveness in financial markets, improve efficiency in the allocation of credits, reduce information asymmetry, and increase the volume of lending [67, 68].

Financial institutions often face the problem of inconsistent information shared by small farmers, which not only increases transaction costs but also lending risks. The exchange of accurate information would not only solve the supply gap problems of an emerging industry, but also increase the financial support of small organic farmers [62]. Further, the exchange of accurate information will motivate financial institutions to tackle the problem of asymmetric information by utilizing this network and designing innovative financial products for value chain actors [29]. According to Minkyun, information sharing among the value chain actors build better partnerships, and promote producer-buyer integration, thereby saving time and cost for innovative and make agile value chain finance [69].

### Contractual governance

Contract governance refers to the degree to which a contractual partnership is regulated by a formal contract specifying formal rules, responsibilities, and duties [70,71]. Governance prohibits the value chain members from pursuing their own interests in various ways. This is because misinformation or fraud is considered a breach of contract, and such behaviors are not acceptable. The contractual governance, therefore, sets out ways to resolve disputes, which in turn increase transaction efficiency [72].

Contractual governance of the food value chain is on the rise [73]. It supports innovation-based coordination and strict collaboration among participants. This collaborative relationship among value chain partners, according to Anna Grandori [74], regulates transactions and pool resources, and hence, procedural for the innovation. CG also has the capability to strengthen the interdependence relationship among value chain partners [75], consequently, improve the collaboration and swifter the financial flow [76].

### Information and communication technology

In recent decades, the growing trend of ICT in the financial industry has been a well-documented fact. ICT platforms can help banks by reaching even the most dispersed areas through adequate communication infrastructures [77]. In many Asian countries such as Pakistan, mobile banking is taking off and has already become one of the “new and fast-developing spaces at the convergence of technology and financial services”‘ [78]. It saves from the exploitative behavior of the middle-men, who use the prevailing information gap and claim a relatively high interest rate [19,79]. ICT will improve the creditworthiness of smallholders, thereby strengthening the partnerships among value chain actors that reduce the cost of interaction among stakeholders and the risk associated with VCF [80, 81].

The financial and information flow alongside the value chain improves effective production, which will encourage the banking sector for innovation and product development. According to Zhao et al. [82] the use information technologies improves the innovativeness of financial services, lowers costs, controls risks, and makes agile financial flow.

### Innovativeness

Innovativeness refers to a system in which a value chain collaborates with its partners and introduces new services or products to enhance customer satisfaction [83]. By carefully managing producers and buyers’ relationships, financial institutions can greatly improve their ability by strengthening the process of product innovation [84].

Innovativeness is associated with the timely delivery of new products that provide higher value to customers. With the rapid changes in the technologies and customer trends, business organizations should take full advantage of market demands by developing innovative products. In general, financial institutes with a high degree of innovativeness can adapt to changes in the business environment to ensure the value chain competitiveness [85].

### Agility

Agility is described as relevancy, which is “the ability to maintain focus on the changing needs of customers,” accommodation is “the ability to respond to unique customer requests,” and flexibility, is “the ability to adapt to unexpected circumstances”[86]. Therefore, agility in a value chain allows financial institutes to respond quickly to customer needs in order to achieve competitiveness. Thus, agility in value chain finance is defined as the degree of swiftness with which value chains respond to customers financing needs. Effective formal credit accelerates financial flow within value chain and reduces the role of informal moneylenders [87] e.g. middlemen. To accelerate the VCF, agility represents the speed that increases product customization, improves delivery performance, and reduces development time. Thus, we operationalize agility in the VCF as the speed at which a value chain can improve its competitive position with respect to financial product delivery speed and innovation [86].

### Value chain competitiveness

According to Rao and Holt [88], competitiveness at value chain level means, improved efficiency, productivity improvement, reduce risks and cost savings. It has also been recognized that the range of services, including trust and cooperation, product innovation, information sharing, and agility increase the flow of financial services that ultimately enhance the competitiveness of the value chain [89]. Thus, for financial institutions, the VCF creates the motivation to craft innovative financial products that best fit to the needs of smallholders [25]. Accordingly, increase level of financial services to smallholders, give access to high-value markets and enhance the value chain competitiveness [90]. Therefore, to achieve competitiveness in organic food value chain finance, agility and innovativeness can be very useful, to respond quickly, to the financial needs of smallholders.

Therefore, the following hypothesis are considered:

**Table:** 

*H*_1*a*_:	There is positive relationship between IT integration of value chain members and innovative financial product development.
*H*_1*b*_:	There is positive relationship between IT integration of value chain members and value chain finance agility.
*H*_2*a*_:	Trust between value chain partners positively related to innovative financial product development.
*H*_2*b*_:	Trust between value chain partners positively related to value chain finance agility.
*H*_3*a*_:	Information sharing between value chain partners positively associated to innovative financial product development.
*H*_3*b*_:	Information sharing between value chain partners positively associated value chain finance agility.
*H*_4*a*_:	Contractual governance between value chain partners positively associated to innovative financial product development.
*H*_4*b*_:	Contractual governance between value chain partners positively associated to value chain finance agility.
*H*_5*a*_:	The use of ICT between value chain partners positively associated to innovative financial product development.
*H*_5*b*_:	The use of ICT between value chain partners positively associated to value chain finance agility.
*H*_6*a*_:	Financial innovation in products and services positively related to organic food value chain competitiveness.
*H*_6*b*_:	Value chain finance agility is positively related to organic food value chain competitiveness.
*H*_7_:	Innovation in financial products and agility in value chain finance positively related to organic food value chain competitiveness.

## Research methods

### Sampling and data collection

**Fig 3:**
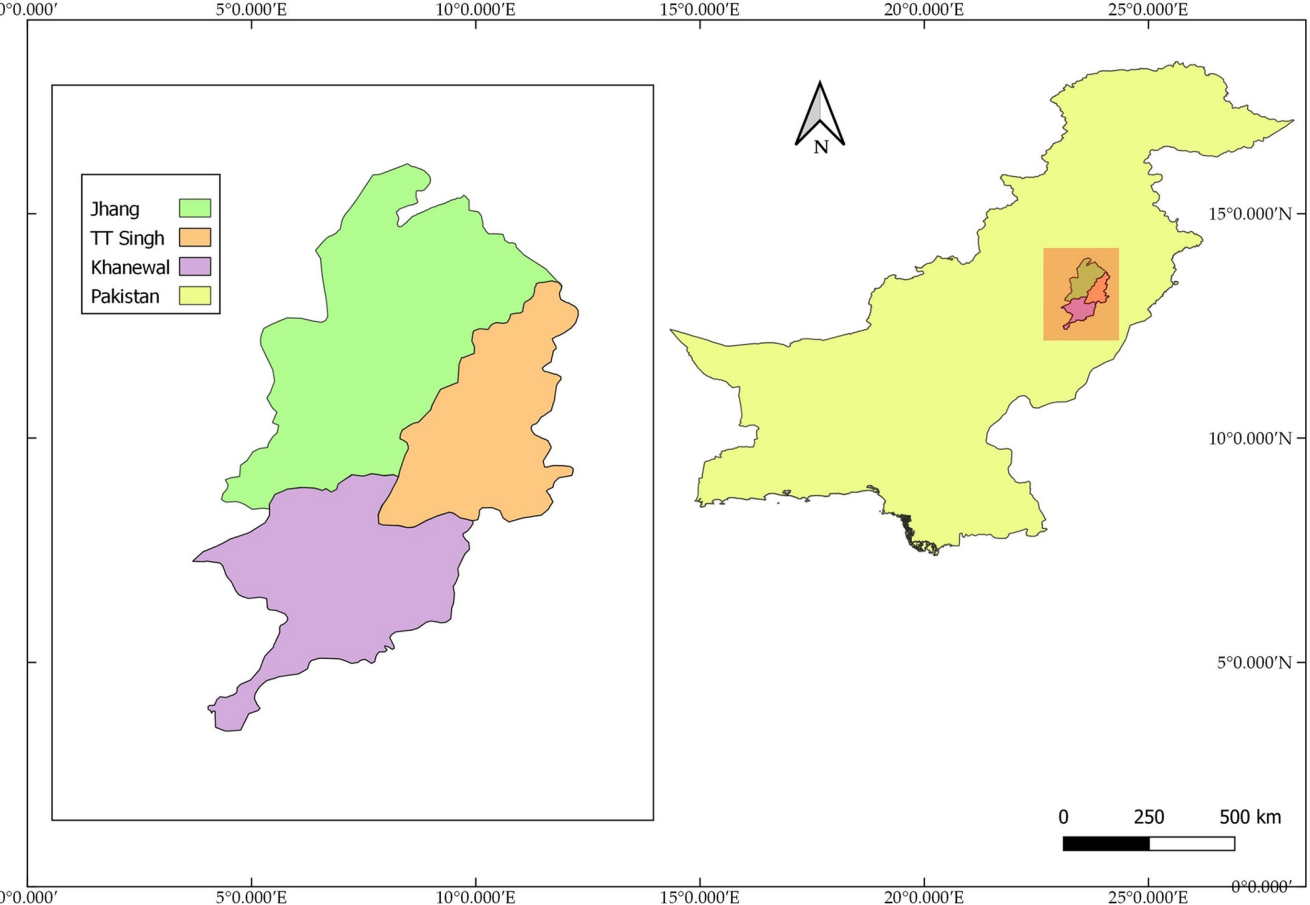
Study area.

**Fig 4:**
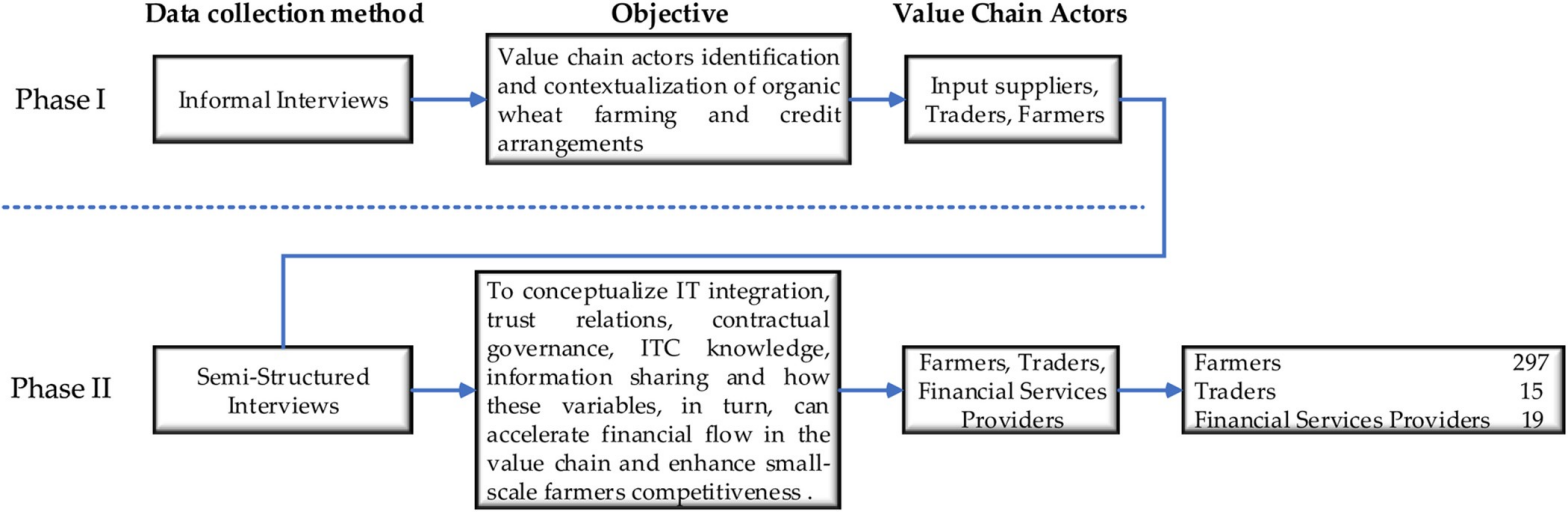
Data collection methods.

The present study was conducted in Central Punjab, Pakistan in three districts of Jhang, Toba Tek (TT) Singh, Khanewal, (Fig 3). The research was conducted in two phases, as shown in (Fig 4). The first phase was informal discussions with several organic farmers, input suppliers, and traders to decide how the input and support services for producers are generally organized in and around the municipalities. Such discussions have been used to focus on three major value chain actors engaged in smallholder credit: producers, investors or retailors, and lending institutions. All three districts are representative of the farming communities in central Punjab with an organic wheat production history. For data collection, we selected farmers through a researcher. We chose traders based on their experience in handling credits with farmers. The lending institutions were selected on the basis of discussions with the farmers, based on the kind of creditors they usually engaged with.

In the second phase, we conducted semi-structured interviews to collect data. These interviews were conducted in the local language for the convenience of organic wheat farmers, ranging from 100 to 110 producers in each district. Fifteen traders were interviewed, each selected based on the criteria for credit experience history. Two types of financial service providers were actively engaged: microfinance banks and middlemen/arthi. The topics addressed in the interviews were based on informal conversations as well as literature on credit and value chains. All interviews were conducted with the consent of respondents, and confidentiality was assured.

### Preliminary list of variables

An extensive and thorough review of the scientific literature was carried out to select the variables that emphasize the competitiveness of value chain and make agile the financial flow within the organic food value chain by which risk might be overcome are shown in Table A1 in S1 Appendix. The questionnaire for the interview consisted of two parts. The part one included question about the respondents' personal information, whereas the second section of the questionnaire consisted of information related to preliminary variables, which were divided into eight categories, depending on the nature of the variables: ITI, TRST, CG, IS, ICT, INNOV, AGLTY, and VCC.

### Measures

All measuring objects in the present research have been adapted from relevant literature with slight changes and rephrasing to ensure the contextual accuracy. For each measure, participants assessed their statements of agreement on a seven-point Likert scale ranging from strongly agree (7), neither agree nor disagree (4), or strongly disagree (1). Table A1 in S1 Appendix, comprises a list of the indicators employed in this study. ITI refers to the extent to which various value chain partners communicate, coordinate, and integrate relevant information. To measure the IT integration, a five-item scale was developed after reviewing various literature [91–95]. TRST refers to partners' loyalty and commitment by obeying the rules of transaction and maintaining good attitude and behavior [96]. A five-item scale was used from the study of Doney and Cannon [97] to measure TRST. CG refers to the degree to which a collaborative partnership is regulated by a formal contract specifying formal rules, responsibilities, and duties. A four-item scale was adopted from Cao and Lumineau [70], and Zhou and Poppo [71] to measure the CG. IS refers to the degree to which value chain participants exchange a variety of specific, reliable, and complete financial information in a timely manner. The five-item scale was taken from Cao and Zhang [98] to measure information sharing. ITC focuses on the advancement of rural development through improved information and communication processes by which financial flow accelerates and reduces information asymmetry within the organic food value chain. ITC is measured with five items, adopted from the study of Oladele [99]. Innovativeness is the ability to develop new financial products and services, to interact with customers to fulfill the financing needs. Innovativeness is assessed using five items, adopted from Hurt et al. [100], and Calantone et al. [101]. Agility is defined as the degree of quickness with which financial institutes respond to customers’ financing needs. Thus, agility in value chain finance is measured using six items adopted from Zhang et al. [102] and Swafford et al. [54]. VCC refers to the ability of the smallholder organic farmers to access financing for purchase inputs as well as make investments, to achieve and maintain decent living standards, reinvest in their farms, and continue to provide sustainable food to consumers. To measure the value chain competitiveness, a five-item scale was developed after various literature reviews [11,103–108].

## Partial least square structural equation modeling analysis

The PLS-SEM technique was used to analyze the theoretical model, as shown in Fig 1. We used a two-step modeling method for data analysis, proposed by Anderson and Gerbing [109]. Phase one includes evaluating the measurement model, while phase two measures the structural model (including test of hypothesis). Particularly, the PLS-SEM method was used to conduct the SEM analysis with the help of a statistical program SmartPLS 3.2.9. PLS-SEM is presently recognized and used as the best method for multivariate analysis within social science studies [41]. In addition, to estimate a more complex model and estimate mediation effects, PLS-SEM is more accurate to account for measurement errors. This approach's strength lies in its flexibility to deal with complex models relative to other SEM methods [110].

Table A2 in S1 Appendix, describes comprehensive results of descriptive statistics such as, skewness, kurtosis, mean and standard deviation. According to Hair et al. [111], skewness and kurtosis values range between -1 and +1, are acceptable for being normally distributed data. The results of our study demonstrated that data were normally distributed.

### Evaluation of the outer (reflective measurement) model

The purpose of the reflective model is to analyze the reliability and validity of the observed variables together with latent variables [112]. The construct of the conceptual model can be assessed in two ways: composite reliability (CR) and Cronbach alpha (α). For both reliability criteria, the rule of thumb is that the values must be above 0.70 and lower than 0.95 [113]. In Table 1, the measurements of Cronbach Alpha (α) and Composite Reliability (CR), of the PLS-PM measurement model are provided. Cronbach’s alpha and Composite Reliability values ranges between 0 and 1; value closer to one indicates a higher internal consistency, values closer to zero indicate a lower internal consistency. The sixth and seventh column in Table 1, showed that the values of α and CR were above 0.9, indicate nearly perfect reliability of the measures. Convergent validity is the extent to which a measure correlates positively with alternative measures of the same construct. It can be measured through Average Variance Extracted (AVE). An adequate AVE is 0.50 or higher because it demonstrates that the construct explains more than 50% of the variance of its items [113]. As shown in Table 1, the AVE values were more than 0.7 (70%); therefore, convergent validity was established for this conceptual model.

**Table 1:** Convergent validity and reliability test of conceptual model.

Construct	Mean	Standard Deviation	Item	Loadings	Cronbach Alpha	CR	AVE
VCC^∅^	4.155	1.103	VCC_***i***_	0.873	0.925	0.943	0.769
			VCC_***j***_	0.881			
			VCC_*k*_	0.881			
			VCC_*l*_	0.890			
			VCC_***m***_	0.858			
INNOV^∅^	3.413	1.301	INNOV_***i***_	0.938	0.936	0.934	0.881
			INNOV_***j***_	0.958			
			INNOV_*k*_	0.946			
			INNOV_*l*_	0.924			
			INNOV_***m***_	0.925			
AGLTY^∅^	3.610	0.974	AGLTY_***i***_	0.839	0.920	0.938	0.714
			AGLTY_***j***_	0.883			
			AGLTY_*k*_	0.830			
			AGLTY_*l*_	0.820			
			AGLTY_***m***_	0.846			
			AGLTY_***n***_	0.852			
ITI^∅^	3.914	1.168	ITI_***i***_	0.909	0.947	0.926	0.852
			ITI_***j***_	0.924			
			ITI_*k*_	0.920			
			ITI_*l*_	0.908			
			ITI_***m***_	0.953			
TRST^∅^	4.242	1.369	TRST_***i***_	0.903	0.916	0.906	0.852
			TRST_***j***_	0.938			
			TRST_*k*_	0.921			
			TRST_*l*_	0.917			
			TRST_***m***_	0.934			
IS^∅^	4.197	1.177	IS_***i***_	0.899	0.912	0.938	0.791
			IS_***j***_	0.881			
			IS_*k*_	0.868			
			IS_*l*_	0.910			
			IS_***m***_	0.899			
CG^∅^	4.392	1.293	CG_***i***_	0.908	0.940	0.937	0.849
			CG_***j***_	0.942			
			CG_*k*_	0.917			
			CG_*l*_	0.917			
ICT^∅^	3.317	1.079	ICT_***i***_	0.890	0.946	0.918	0.822
			ICT_***j***_	0.923			
			ICT_*k*_	0.886			
			ICT_*l*_	0.903			
			ICT_***m***_	0.929			

Note: ∅ = Latent Construct.

ITI^∅^ = IT Integration; TRST^∅^ = Trust; IS^∅^ = Information Sharing; CG^∅^ = Contractual Governance; ICT^∅^ = Information & Communication Technology; INNOV^∅^ = Innovativeness; AGLTY^∅^ = Agility; VCC^∅^ = Value Chain Competitiveness.

*i* = indicator variable 1; *j* = indicator variable 2; *k* = indicator variable 3.

*l* = indicator variable 4; *m* = indicator variable 5; *n* = indicator variable 6.

The next step is to determine the discriminating validity of the constructs, once the reliability of the reflective constructs had been established. Discriminant validity demonstrates whether the constructs in the model are highly correlated among them or not. Heterotrait-Monotrait Ratio (HTMT) was used to evaluate discriminant validity as recommended by Henseler and his research fellows [114]. The results in Table 2 demonstrate that HTMT values are significantly lower than the conservative threshold (HTMT_0.85_), meaning that all variables in the conceptual model have discriminant validity.

**Table 2:** Discriminant validity test of the financing model: Heterotrait-Monotrait Ratio (HTMT).

	CG^∅^	ITI^∅^	ICT^∅^	IS^∅^	INNOV^∅^	TRST^∅^	AGLTY^∅^
**Contract Governance**							
**IT Integration**	0.563						
**Information & Communication Technology**	0.313	0.295					
**Information Sharing**	0.449	0.431	0.288				
**Innovativeness**	0.595	0.547	0.534	0.522			
**Trust**	0.536	0.510	0.342	0.453	0.551		
**Agility**	0.641	0.615	0.477	0.531	0.658	0.605	
**Value Chain Competitiveness**	0.607	0.454	0.364	0.456	0.579	0.532	0.643

Note: ITI^∅^ = IT Integration; TRST^∅^ = Trust; IS^∅^ = Information Sharing; CG^∅^ = Contractual Governance; ICT^∅^ = Information & Communication Technology; INNOV^∅^ = Innovativeness; AGLTY^∅^ = Agility; VCC^∅^ = Value Chain Competitiveness.

Thus, the proposed theoretical model was deemed appropriate, with verification of sufficient reliability, convergent validity, and discriminating validity.

### Evaluation of the inner (structural) model

According to J. Hair et al [115] PLS-structural equation modelling is always the preferred SEM method when the research objective is to prediction of relationships between the constructs. The evaluation of the inner (structural) model included observing the predictive relevancy of the proposed model and the relationships between the constructs. The major criteria for assessing the inner structural model are the coefficient of determination (R^2^), path coefficient (β value), and T-statistic value. The other valuable methods used for measuring structural model included the predictive relevance of the model (Q^2^), effect size (ƒ^2^), and goodness-of-fit (GOF) index.

### Path coefficients and p-values

In the regression analysis, the path coefficients and the standardized β coefficient were similar. The significance level of the hypothesis was checked using the β value. β is defined as the anticipated variance in the endogenous constructs for a unit variation in the exogenous construct(s). The greater the β-value, the higher the significant impact on the endogenous latent structure [110] (Table 3).

**Table 3:** Path coefficients and p-values of model.

Hypothesized Path	Standardized Beta	t-value
Innovativeness ⇒ Value Chain Competitiveness	0.294***	6.099
Agility ⇒ Value Chain Competitiveness	0.413***	6.965
IT Integration ⇒ Innovativeness	0.169*	3.708
IT Integration ⇒ Agility	0.228**	5.025
Trust ⇒ Innovativeness	0.156*	3.284
Trust ⇒ Agility	0.197*	3.965
Information Sharing ⇒ Innovativeness	0.184*	3.504
Information Sharing ⇒ Agility	0.156*	3.724
Contractual Governance ⇒ Innovativeness	0.236**	5.494
Contractural Governance ⇒ Agility	0.250**	5.299
Information & Communication Technology ⇒ Innovativeness	0.293**	6.089
Information & Communication Technology ⇒ Agility	0.201**	4.538

Note:

“* significant for p≤0.05.”

“** significant for p≤0.01.”

“*** significant for p≤0.000.”

**Fig 5:**
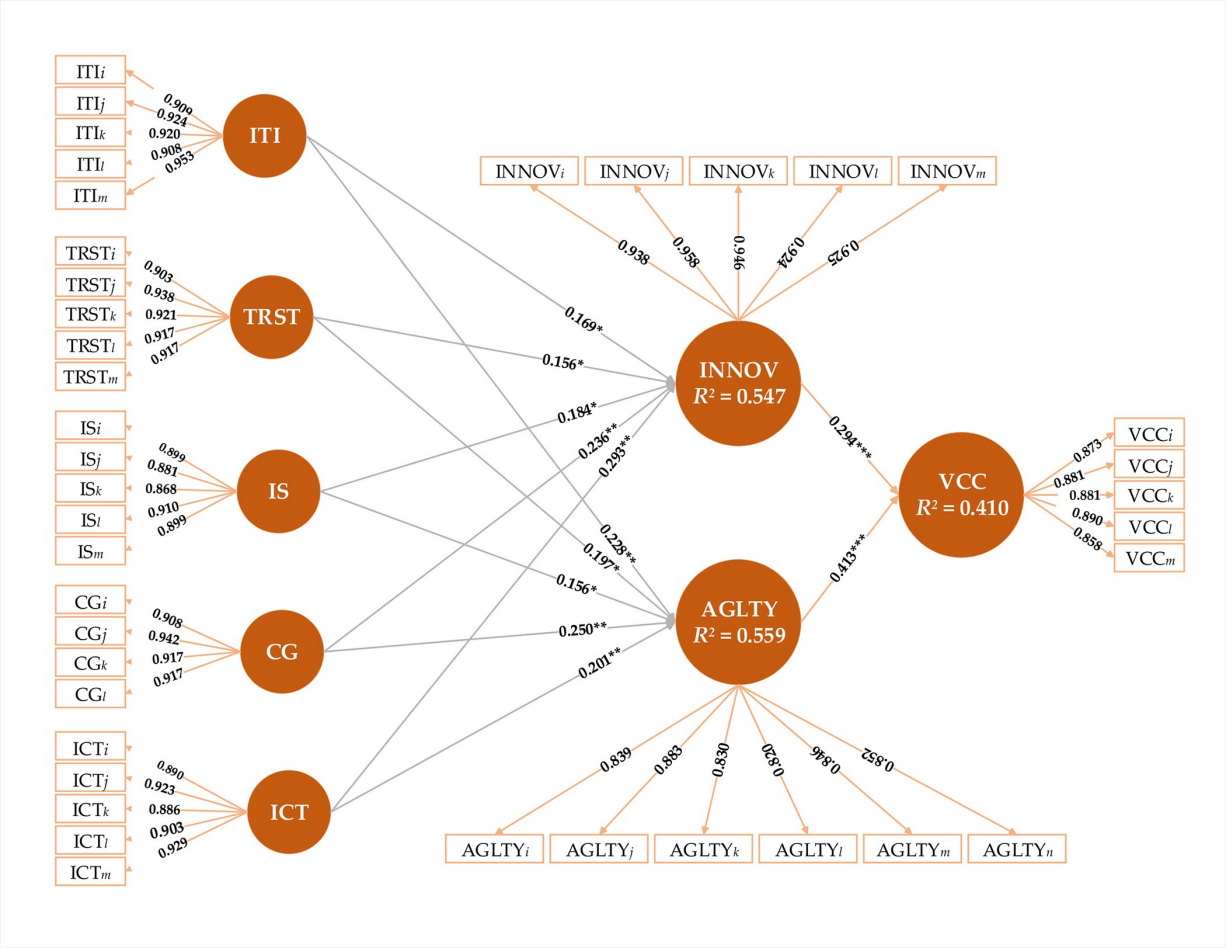
Results of structural equation model analysis.

The path coefficients of the structural model were used to test the proposed hypotheses. All twelve structural path coefficients demonstrated at least a p-value less than 0.05. The findings of the present study indicate that all the proposed hypotheses are verified and accepted. More precisely, ITI has a significant and positive effect on INNOV (*β* = 0.169, T = 3.708, *p* < 0.000) and AGLTY (*β* = 0.228, T = 5.025, *p* < 0.000). Therefore, *H*_1*a*_ and *H*_1*b*_ are supported. Trust positively influences INNOV (*β* = 0.156, T = 3.284, *p* < 0.000) and AGLTY (*β* = 0.197, T = 3.965, *p* < 0.000), lending support to *H*_2*a*_ and *H*_2*b*_. *H*_3*a*_ and *H*_3*b*_, which proposes that IS directly affects INNOV (*β* = 0.184, T = 3.504, *p* < 0.000) and AGLTY (*β* = 0.156, T = 3.724, *p* < 0.000) are also accepted. As expected, CG has a significant impact on INNOV (*β* = 0.236, T = 5.494, *p* < 0.000) and AGLTY (*β* = 0.250, T = 5.999, *p* < 0.000), thus supporting *H*_4*a*_ and *H*_4*b*_. As expected, ITC has a positive impact on INNOV (*β* = 0.293, T = 6.089, *p* < 0.000) and AGLTY (*β* = 0.201, T = 4.538, *p* < 0.000), thus supporting *H*_5*a*_ and *H*_5*b*_. Our results also support previous research in relation to INNOV (*β* = 0.294, T = 6.099, *p* < 0.000) and AGLTY (*β* = 0.413, T = 6.0965, *p* < 0.000) have a positive effect on competitiveness, thereby supporting *H*_6*a*_ and *H*_6*b*_. The findings in Table 3, and (Fig 5), demonstrate that value chain competitiveness could be acquired from agility and innovative financial product development.

### Measuring the values of R^2^, Q^2^, and f^2^

The coefficient of determination (R^2^) values were determined for all endogenous constructs to test the quantity of variance described in the dependent variables seen as the structural model's predictive power. According to Hair et al. [39], the R^2^ value of 0.75 is substantial, 0.50 is moderate, and 0.25 is weak. As shown in Table 4, all the independent variables (ITI, TRST, IS, CG, ICT) demonstrated a high variance, that is, 54.7% within INNOV (*R*^2^: INNOV = 0.547). These five independent latent constructs explain the variance of 55.9% within agility (R2 VCA = 0.559). Whilst, INNOV and agility explained the moderate variance i.e. 40.7% in the value chain competitiveness (R^2^: VCC = 0.407).

**Table 4:** Predictive relevancy and predictive accuracy of conceptual model.

Endogenous Construct	*R*^*2*^	*Q*^*2*^	Relationship	*f*^*2*^	Effect size
Value Chain Competitiveness	0.407	0.307	INNOV^∅^ ⇒ VCC^∅^	0.089	Weak
			AGLTY^∅^ ⇒ VCC^∅^	0.176	Moderate
Innovativeness	0.547	0.475	ITI^∅^ ⇒ INNOV^∅^	0.039	Weak
			TRST^∅^ ⇒ INNOV^∅^	0.034	Weak
			IS^∅^ ⇒ INNOV^∅^	0.055	Weak
			CG^∅^ ⇒ INNOV^∅^	0.075	Weak
			ICT^∅^ ⇒ INNOV^∅^	0.162	Moderate
Agility	0.559	0.393	ITI^∅^ ⇒ AGLTY^∅^	0.074	Weak
			TRST^∅^ ⇒ AGLTY^∅^	0.056	Weak
			IS^∅^ ⇒ AGLTY^∅^	0.041	Weak
			CG^∅^ ⇒ AGLTY^∅^	0.086	Weak
			ICT^∅^ ⇒ INNOV^∅^	0.078	Weak

Note: ITI^∅^ = IT Integration; TRST^∅^ = Trust; IS^∅^ = Information Sharing; CG^∅^ = Contractual Governance; ICT^∅^ = Information & Communication Technology; INNOV^∅^ = Innovativeness; AGLTY^∅^ = Agility; VCC^∅^ = Value Chain Competitiveness.

***R***^2^ = Coefficient of Determination.

***Q***^2^ = Predictive relevance of Model.

***f***^2^ = effect size.

Predictive relevance of Model (Q^2^) was then assessed using blindfolding procedures [116], whereas cross-validated redundancy was performed as suggested by Chin [110]. In the SEM, for a specific endogenous latent construct, the Q^2^ values measured should be greater than zero. From Table 4, it can be seen that the Q^2^ values for value chain competitiveness, INNOV and AGLTY were 0.307, 0.457, and 0.393, respectively, which are statistically acceptable for predictive relevance. To check the impact of each latent exogenous construct on the latent endogenous constructs, the effect size (f^2^) was assessed (Table 4). According to Hair et al. [117] the values of effect size (f^2^) 0.02 demonstrate a weak effect, 0.15 is a moderate effect, and 0.35 is a substantial effect. According to these guidelines, Table 5 demonstrates that ITI, TRST, IS, and CG have a weak effect on both INNOV and AGLTY, whereas ICT has a moderate effect on INNOV. Innovativeness exhibits a weak effect on value chain competitiveness, while AGLTY has a moderate effect on value chain competitiveness. In conclusion, the R^2^, Q^2^, and f^2^ test results suggest that the findings drawn from this study are relatively robust.

**Table 5:** Calculation of goodness of fit index.

Construct	AVE	*R*^*2*^
IT Integraton^∅^	0.852	
TRUST^∅^	0.852	
Information Sharing^∅^	0.791	
Contractual Governance^∅^	0.849	
Information and Communication Technology^∅^	0.822	
INNOVATIVENESS^∅^	0.881	0.547
AGILITY^∅^	0.714	0.559
Value Chain Competitiveness^∅^	0.769	0.407
**Average Values**	**0.8163**	0.5043
***AVE* X *R***^***2***^	**0.4116**	
**GoF**	**0.642**	

Note: ∅ = Latent Construc.

AVE = Average Variance Extracted, ***R***^2^ = Coefficient of Determination, *GoF* = *Goodness of Fit Index*.

### Goodness-of-fit index calculation

Partial least square structural equation modeling does not focus on the fit model. Nonetheless, Tenenhaus et al. [116] proposed the GoF as a means to validate a PLS path model globally. A good fit model demonstrates that a model is parsimonious and plausible [118]. It is calculated using the average communality (AVE values) and the average R^2^ value(s). On the basis of formula, proposed by Tenenhaus et al. [116] the value of GOF = 0.642 demonstrate that the fit index is sufficiently good to support the validity of the global model. (Table 5). By using Eq (1), the model’s goodness of fit is calculated as follows [116]:

$$\mathrm{GoF}=\sqrt{average\;communality\times average\;R^{2}}$$ (1)

### Standardized root mean square residual (SRMR)

SRMR is a measure of the mean absolute value of the correlation residuals. When values of SRMR = <0.08, the research model has a good fit [119] however, a lower SRMR is considered to be a better fit. Table 6, demonstrates that the conceptual model’s SRMR was 0.045, which showed that the conceptual model had a good fit.

**Table 6:** Goodness of fit index summary.

	Estimated Model
SRMR	**0.045**
d_ULS	1.610
d_G	0.631
Chi-Square	1200.916
NFI	0.913

Note: *SRMR* = Standardized root mean square redidual, *d*_*ULS*_ = squared Euclidean distance*d*.

*d*_*G* = geodesic distance, *NFI* = Normed fit Index.

In accordance with the complete evaluation of both inner (structural) model and outer (measurement) model, it was determined that all of the hypotheses were statistically significant and hence were all verified.

**Fig 6:**
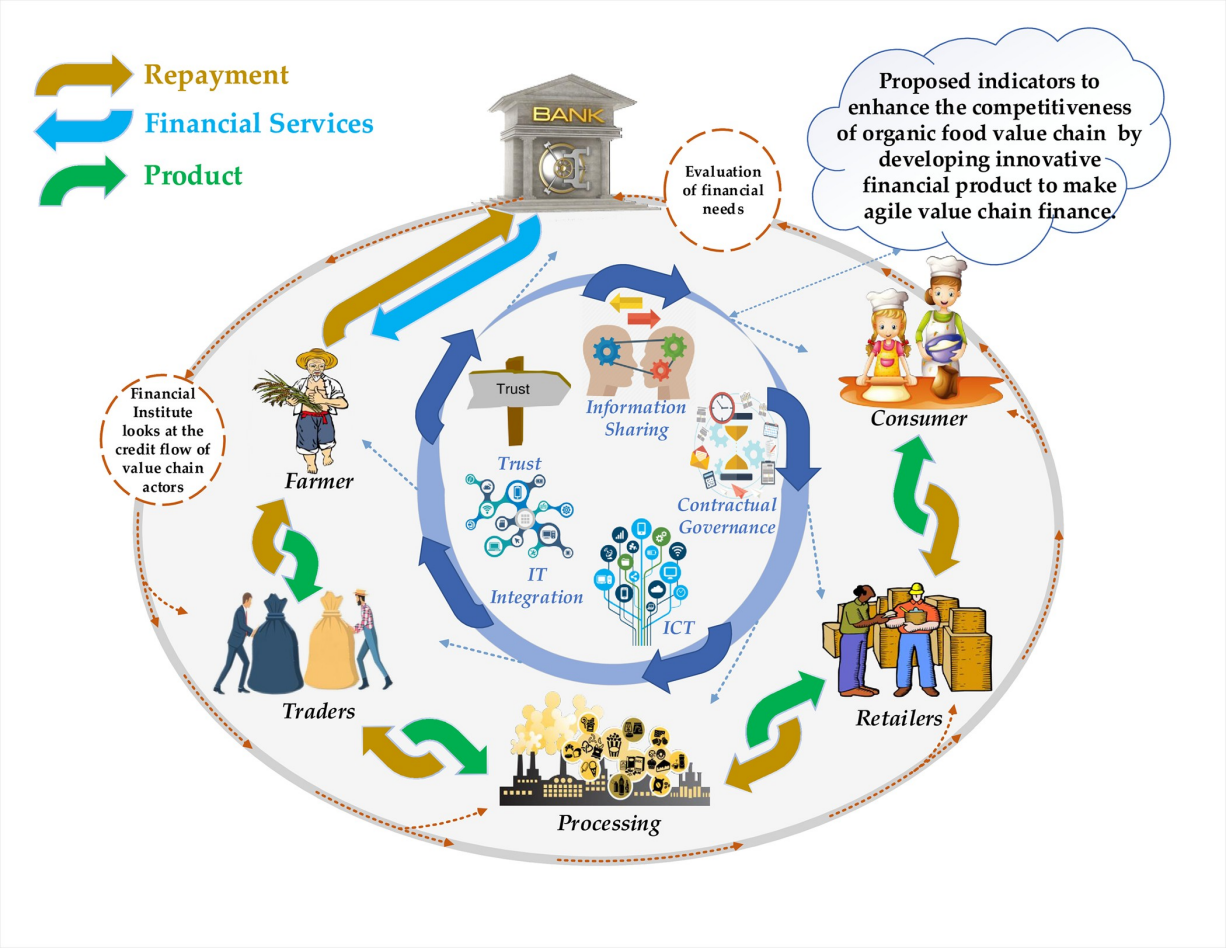
The graphically representation of smallholder’s credit access and organic food value chain finance.

The contribution of organic food value chains is graphically presented in (Fig 6). The illustration shows how the propose indicators enhance the competitiveness of organic food value chain by developing innovative financial product to make agile value chain finance. Through IT integration of partners, trust-based relations, governance arrangements, ITC-based knowledge, and accurate information sharing among the value chain partners that would encourage the financial institutes for the development of innovative financial products to accelerate the financial flow and to achieve competitiveness.

## Discussion & conclusion

In this study, a number of theoretical contributions were made. The key contribution of this study was to develop a conceptual model to address the financial needs of smallholders and mitigate the financial risk by organizing value chain actors through information technology and governance arrangements. The present study applied an advanced multivariate analysis technique of the PLS path model to examine and validate the conceptual model. PLS-SEM is a very advanced technique for developing and evaluating a complex model and social science researchers must incorporate the latest techniques to manage their current and future studies. Moreover, this study fills a gap in the literature on organic farming by investigating the impact of ITI, TRST, CG, IS and ICT on value chain competitiveness through the mediating effects of INNOV and AGLTY. In this study, we explore ITI, TRST, CG, IS, and ICT can facilitate AGLTY and INNOV, and thus have a substantially positive effect on value chain competitiveness.

The study demonstrates that ITI among value chain members significantly associated to innovative product development and value chain finance agility. IT integration between value chain actors increase smooth flow of information, thereby strengthening the trustworthiness. IT based integration of value chain actors can better informed about the product prices and will give easy access to the market. The financial institutes can utilize this network to reduce the information asymmetry, and overcome the problem of financial risk. Apart from this, financial institute design innovative product that best fit for smallholders and value chain actors. Our results suggest that IT integration rapidly adopt the changes through a dynamic network of linkages of value chain actors to respond financial needs. Thus, integration of value chain actors through information technology not only increase the creditworthiness of smallholder but also enhance financial flow and improve competitiveness of organic food value chain. In line with our study Chen et al. [30] and Chia Tan et al. [120] found that IT integration between value chain actors provide the potential to continually innovate the products to catalyze finance and investment. The results from our research indicate that trust significantly influences INNOV and value chain finance agility. The better trust among value chain members in terms of close relationships, being honest and trustworthy, etc., will reduce the uncertainty and risk for creditors. The findings of the study are based on the argument of Miller and Jones [25] who claimed that trust between the producer and the buyer can drive innovation in value chain finance and continue to mitigate risks for lending institutes. IS generates frequent communication between producers and buyers. Frequent communication with farmers has a positive impact on the VCF for both producers and buyers. Prior studies found that information sharing reconcile information asymmetry issues between farmers and lending institutes [62] which leads to craft of innovative products and value chain finance agility [69]. IS provides an opportunity for financial service providers by making decisions based on right information. Thus, IS helped financial institutes to make quick decisions about changes in the market by having frequent communications with value chain actors. CG is an efficient instrument for connecting low-income growers into value chains and increasing the income of small-scale producers, thus reducing transaction costs. In this study, CG has a significant and positive impact on INNOV and value chain finance agility. Thus, acting as communication tools for transmission of information from one actor to another actor to reduce uncertainty and risks. This will strengthen the codependent relationships, and maintain relationships between smallholder, value chain partners and financial institutes. In accordance with our findings, Enquist et. al [75] found that contractual governance develops innovative products and increase inter-organizational performance. Governance arrangements were also found to have direct effects on agility that means to respond quickly [76] according to customer needs. ICT has also been positively related to agility and INNOV. The results of our study suggested that most of the respondents were well versed with information technology, particularly internet and mobile phones. Increased levels of digitalization, such as mobile services, play an important role with respect to the development of the VCF efficiency and cost-effectiveness. This fact about the positive role of ICT was also revealed by Altenbuchner et al. [19] and Aldosari et al. [79] in which they proved that the use of ICT has the ability to save from the exploitation from the middlemen who claimed a relatively high interest rate. The findings of the present study are also in accordance with Ali and Kumar [80], Oladele [99] and, Salampasis and Theodoridis [121] in which they proved that through the effective use of ICT the value chain members strengthen partnerships, reduces transaction costs, and financial risks in value chain finance. Furthermore, the results of our research revealed that AGLTY and INNOV significantly affect value chain competitiveness. Higher the INNOV in the form of trying new ideas and ways for financial product development, better the competitiveness of the organic food value chain. The findings of the present study support the results of Ngenoh et al. [90] and Dubey et al. [122] that have explored usefulness of agility and innovativeness in responding quickly to the financial needs of value chain actors, in the rapidly changing environment and gaining higher competitiveness.

## Conclusion

This study set up a conceptual model in the organic food value chain to examine the mediating role of AGLTY and INNOV, including aspects such as ITI, TRST, CG, ICT, and IS, which can accelerate financial flow and enhance value chain competitiveness. The present study sheds light on financial constraints faced by small-scale organic wheat farmers in developing countries such as Pakistan. As such, we conclude that over many years’ financial constraints and the exploitative role of middlemen would be overcome through the ITI, trust relations, CG, ITC-based knowledge, and IS among the value chain partners that would encourage the development of innovative financial products to accelerate the financial flow and to achieve competitiveness. To address these barriers, changes at the farm and market level as well as among financial service providers and farmers might be required, which would only be possible through trust-based relations and governance arrangements. However, farmers’ trustworthy relations with value chain partners, are needed to positively harness innovative product development for swifter value chain finance. In the last, but not least, this theoretical model should not be viewed as a quick fix but as a process of test and learning.

## Supporting information

S1 Dataset A CSV file for “S1 Dataset” used for data analysis in the present study. (RAR)

S1 Appendix [123,124]. (DOCX)
