# Supplementary material for: Application of structural equation modelling to develop a conceptual model for smallholder’s credit access: The mediation of agility and innovativeness in organic food value chain finance
Source: PLoS One. 2020 Aug 4;15(8):e0235921. doi: 10.1371/journal.pone.0235921 (PMC7402512; doi:10.1371/journal.pone.0235921)
Supplement: S1 Appendix — (DOCX) [file pone.0235921.s002.docx]

**Appendix A**

**Table A1.** The list of preliminary variables for conceptual model

| **Construct** | **Indicators** | **Description/Keywords** | **References** |
| --- | --- | --- | --- |
| $\mathrm{IT}I^{\emptyset}$ | $\mathrm{ITI}_{\boldsymbol{i}}$ | ITI Reduced transaction cost. | (91) (92) (93) (94) (95) |
|  | $\mathrm{ITI}_{\boldsymbol{j}}$ | ITI Increase cooperation and coordination. |  |
|  | $\mathrm{ITI}_{k}$ | ITI helpful for new product development. |  |
|  | $\mathrm{ITI}_{l}$ | ITI significantly impact on value chain. innovation. |  |
|  | $\mathrm{ITI}_{\boldsymbol{m}}$ | ITI agile the delivery of financial products. |  |
| $\mathrm{TRST}^{\emptyset}$ | $\mathrm{TRST}_{\boldsymbol{i}}$ | value chain partners keep promises. | (97) |
|  | $\mathrm{TRST}_{\boldsymbol{j}}$ | We believe the information that our partners provide |  |
|  | $\mathrm{TRST}_{k}$ | value chain partners are trustworthy. |  |
|  | $\mathrm{TRST}_{l}$ | When making important decisions, our partners consider welfare of the value chain. |  |
|  | $\mathrm{TRST}_{\boldsymbol{m}}$ | financial institute keeps our best interests for product development. |  |
| $\mathrm{IS}^{\emptyset}$ | $\mathrm{IS}_{\boldsymbol{i}}$ | Value chain partners exchange relevant information. | (98) |
|  | $\mathrm{IS}_{\boldsymbol{j}}$ | Value chain partners exchange complete information. |  |
|  | $\mathrm{IS}_{k}$ | Value chain partners exchange timely information. |  |
|  | $\mathrm{IS}_{l}$ | Value chain partners exchange accurate information. |  |
|  | $\mathrm{IS}_{\boldsymbol{m}}$ | Value chain partners exchange confidential information. |  |
| $\mathrm{CG}^{\emptyset}$ | $\mathrm{CG}_{\boldsymbol{i}}$ | We have a specific, well-defined agreement with our value chain partners. | (123) |
|  | $\mathrm{CG}_{\boldsymbol{j}}$ | We have customized agreements that detail the obligations. |  |
|  | $\mathrm{CG}_{k}$ | We have detailed contractual agreements specifically designed for value chain partners. |  |
|  | $\mathrm{CG}_{l}$ | Our contracts precisely define what will happen in case of unexpected events. |  |
| $\mathrm{ICT}^{\emptyset}$ | $\mathrm{ICT}_{\boldsymbol{i}}$ | ICT improves financial flow. | (99) |
|  | $\mathrm{ICT}_{\boldsymbol{j}}$ | ICT increases information flow and knowledge on farming activities. |  |
|  | $\mathrm{ICT}_{k}$ | ICT improves access to organic food markets. |  |
|  | $\mathrm{ICT}_{l}$ | ICT reduces cost of interaction among stakeholders. |  |
|  | $\mathrm{ICT}_{\boldsymbol{m}}$ | ICT strengthens the partnerships among value chain partners. |  |
| $\mathrm{INNOV}^{\emptyset}$ | $\mathrm{INNOV}_{\boldsymbol{i}}$ | Financial Institute with value chain partners introduces new products and services | (124) (101) |
|  | $\mathrm{INNOV}_{\boldsymbol{j}}$ | Financial Institute with value chain partners has rapid new product development. |  |
|  | $\mathrm{INNOV}_{k}$ | Financial Institute with value chain partners innovates frequently. |  |
|  | $\mathrm{INNOV}_{l}$ | We frequently try out new ideas in the value chain finance context. |  |
|  | $\mathrm{INNOV}_{\boldsymbol{m}}$ | We often introduce new ways of servicing the value chain. |  |
| $\mathrm{AGLTY}^{\emptyset}$ | $\mathrm{AGLTY}_{\boldsymbol{i}}$ | Speed in reducing development cycle time. | (54) |
|  | $\mathrm{AGLTY}_{\boldsymbol{j}}$ | Speed in increasing frequencies of new product introduction. |  |
|  | $\mathrm{AGLTY}_{k}$ | Speed in increasing levels of product customization. |  |
|  | $\mathrm{AGLTY}_{l}$ | Speed in improving customer service. |  |
|  | $\mathrm{AGLTY}_{\boldsymbol{m}}$ | Speed in improving delivery reliability. |  |
|  | $\mathrm{AGLTY}_{\boldsymbol{n}}$ | Speed in improving responsiveness to changing customer needs. |  |
| $\mathrm{VCC}^{\emptyset}$ | $\mathrm{VCC}_{\boldsymbol{i}}$ | The financing of smallholders from a value chain perspective will enhance the productivity of organic food. | (103) (104) (105) (11) (106) (107) (108) |
|  | $\mathrm{VCC}_{\boldsymbol{j}}$ | The financing of smallholders from a value chain perspective will boost economic wellbeing |  |
|  | $\mathrm{VCC}_{k}$ | The financing of smallholders from a value chain perspective will increase economic as well as lending profitability. |  |
|  | $\mathrm{VCC}_{l}$ | The financing of smallholders from a value chain perspective will assist producers to satisfy increased consumer demand |  |
|  | $\mathrm{VCC}_{\boldsymbol{m}}$ | The financing of smallholders from a value chain perspective will increase the livelihoods |  |

$Note: \emptyset= \mathrm{Latent} \mathrm{Construct}$ $\mathrm{IT}I^{\emptyset}$ = IT Integration; $\mathrm{TRST}^{\emptyset}$ = Trust; $\mathrm{IS}^{\emptyset}$ = Information Sharing; $\mathrm{CG}^{\emptyset}$ = Contractual Governance; $\mathrm{ICT}^{\emptyset}$ = Information & Communication Technology; $\mathrm{INNOV}^{\emptyset}$ = Innovativeness; $\mathrm{AGLTY}^{\emptyset}$ = Agility; $\mathrm{VCC}^{\emptyset}$ = Value Chain Competitiveness; $\mathrm{VCF}$ = Value Chain Finance

$i = indicator variable 1$ *;* $j = indicator variable 2$ *;*$k = indicator variable 3$ *;*

$l = indicator variable 4$ *;*$m = indicator variable 5$ *;*$n = indicator variable 6$

**Appendix A**

**Table A2.** Descriptive statistics

|  | **Mean** | **Std. Deviation** | **Skewness** | **Kurtosis** |
| --- | --- | --- | --- | --- |
| $\mathrm{ITI}_{i}$ | 3.86 | 1.216 | -.122 | -.256 |
| $\mathrm{ITI}_{j}$ | 3.91 | 1.331 | -.126 | -.704 |
| $\mathrm{ITI}_{k}$ | 3.95 | 1.276 | -.138 | -.223 |
| $\mathrm{ITI}_{l}$ | 3.82 | 1.226 | .130 | -.106 |
| $\mathrm{ITI}_{m}$ | 4.03 | 1.280 | -.103 | -.552 |
| $\mathrm{TRST}_{i}$ | 4.20 | 1.482 | -.425 | -.853 |
| $\mathrm{TRST}_{j}$ | 4.40 | 1.439 | -.527 | -.591 |
| $\mathrm{TRST}_{k}$ | 4.34 | 1.418 | -.461 | -.520 |
| $\mathrm{TRST}_{l}$ | 4.09 | 1.560 | -.243 | -.090 |
| $\mathrm{TRST}_{m}$ | 4.19 | 1.519 | -.294 | -.921 |
| $\mathrm{IS}_{i}$ | 4.19 | 1.373 | -.341 | -.654 |
| $\mathrm{IS}_{j}$ | 4.24 | 1.385 | -.274 | -.776 |
| $\mathrm{IS}_{k}$ | 4.16 | 1.265 | -.217 | -.557 |
| $\mathrm{IS}_{l}$ | 4.16 | 1.280 | -.163 | -.560 |
| $\mathrm{IS}_{m}$ | 4.24 | 1.349 | -.166 | -.878 |
| $\mathrm{CG}_{i}$ | 4.42 | 1.382 | -.578 | -.448 |
| $\mathrm{CG}_{j}$ | 4.35 | 1.460 | -.548 | -.750 |
| $\mathrm{CG}_{k}$ | 4.36 | 1.408 | -.481 | -.780 |
| $\mathrm{CG}_{l}$ | 4.44 | 1.364 | -.625 | -.287 |
| $\mathrm{ICT}_{i}$ | 3.18 | 1.184 | .764 | -.268 |
| $\mathrm{ICT}_{j}$ | 3.35 | 1.172 | .657 | -.116 |
| $\mathrm{ICT}_{k}$ | 3.39 | 1.121 | .720 | .054 |
| $\mathrm{ICT}_{l}$ | 3.30 | 1.247 | .870 | .230 |
| $\mathrm{ICT}_{m}$ | 3.37 | 1.229 | .919 | .445 |
| $\mathrm{INNOV}_{i}$ | 3.37 | 1.424 | .806 | -.127 |
| $\mathrm{INNOV}_{j}$ | 3.44 | 1.392 | .776 | -.053 |
| $\mathrm{INNOV}_{k}$ | 3.40 | 1.357 | .816 | .002 |
| $\mathrm{INNOV}_{l}$ | 3.50 | 1.331 | .683 | .134 |
| $\mathrm{INNOV}_{m}$ | 3.36 | 1.432 | .834 | -.092 |
| $\mathrm{AGLTY}_{i}$ | 3.63 | 1.089 | -.085 | -.092 |
| $\mathrm{AGLTY}_{j}$ | 3.56 | 1.092 | -.060 | -.047 |
| $\mathrm{AGLTY}_{k}$ | 3.52 | 1.155 | .176 | -.254 |
| $\mathrm{AGLTY}_{l}$ | 3.71 | 1.188 | -.160 | -.159 |
| $\mathrm{AGLTY}_{m}$ | 3.58 | 1.176 | .043 | -.281 |
| $\mathrm{AGLTY}_{n}$ | 3.66 | 1.221 | .000 | -.424 |
| $\mathrm{VCC}_{i}$ | 4.23 | 1.303 | -.419 | -.358 |
| $\mathrm{VCC}_{j}$ | 4.15 | 1.242 | -.335 | -.198 |
| $\mathrm{VCC}_{k}$ | 4.12 | 1.205 | -.401 | -.005 |
| $\mathrm{VCC}_{l}$ | 4.19 | 1.289 | -.285 | -.540 |
| $\mathrm{VCC}_{m}$ | 4.10 | 1.254 | -.249 | -.403 |

$Note: i = indicator variable 1$ *;* $j = indicator variable 2$ *;*$k = indicator variable 3$ *;*

$l = indicator variable 4$ *;*$m = indicator variable 5$ *;*$n = indicator variable 6$
